# Gastric partitioning compared to conventional gastrojejunostomy as palliative surgeries in patients with gastric outlet obstruction: a pairwise and individual patient data meta-analysis

**DOI:** 10.1186/s12957-025-04166-6

**Published:** 2026-01-13

**Authors:** Atef A. Hassan, Mohamed Hamouda Elkasaby, Hazem A. Megahed, Abdorabih Alemam, Mohamed Naroz, Ahmed M. Kandel, Ahmed Fayez Othman, Mohammed Eid Abdelrahman, Mohammed Ali Abdelaty, Boshra Ali El-houseiny, Khaled Mohamed Salamh, Rasha Mohamed Motawea, Hassan Elsayed Younes, Ashraf Ali Abdel Aziz, Ahmed Ali Eldin Taki-Eldin

**Affiliations:** 1 Faculty of Medicine, Al-Azhar University, Cairo, Egypt; 2 General Surgery Department, Faculty of Medicine, Al-Azhar University, Damietta, Egypt; 3 General Surgery Department, Faculty of Medicine, Al-Azhar University for Girls, Cairo, Egypt; 4 General Surgery Department, Faculty of Medicine, Al-Azhar University, Cairo, Egypt; 5 General Surgery Department, Al-Ahrar Teaching Hospital, Zagazig, Egypt; 6 General Surgery Department, Faculty of Medicine, Horus university-Egypt, New Damietta, Egypt

**Keywords:** Gastric outlet obstruction, Palliative surgery, Pancreatic cancer, Stomach-partitioning gastrojejunostomy, Conventional gastrojejunostomy

## Abstract

**Background:**

Gastric outlet obstruction (GOO) complicates unresectable gastric and pancreatic cancers. Conventional gastrojejunostomy (CGJ) is standard but frequently leads to delayed gastric emptying. Stomach-partitioning gastrojejunostomy (SPGJ) mitigates this problem and improves outcomes.

**Methods:**

We conducted a meta-analysis of SPGJ versus CGJ for GOO, searching databases through 25 November 2025. Outcomes were delayed gastric emptying (DGE), major complications, reintervention, 30-day mortality, operative time, Gastric Outlet Obstruction Scoring System (GOOS) scores, length of stay, chemotherapy adherence, and survival. Continuous variables were pooled as mean differences (MD) with 95% CIs; dichotomous variables as relative risks (RR) with 95% CIs. Survival was analyzed using individual patient data reconstructed from Kaplan–Meier curves.

**Results:**

A total of 11 studies comprising 456 patients were included. SPGJ was associated with significantly reduced DGE (RR = 0.24, 95% CI: 0.12–0.47) and postoperative major complications (RR = 0.26, 95% CI: 0.12–0.54) compared to CGJ. No significant differences were found in the need for reintervention (RR = 0.59, 95% CI: 0.21–1.64), short-term mortality (RR = 0.99, 95% CI: 0.42–2.33), or LOS (MD = -1.47 days, 95% CI: -3.10 to 0.16). GOOS scores were comparable between groups. Overall survival was also similar between SPGJ and CGJ (HR = 1.06, 95% CI: 0.66–1.70).

**Conclusions:**

Our meta-analysis shows that SPGJ offers important clinical advantages over CGJ by significantly reducing delayed gastric emptying and postoperative major complications, while demonstrating comparable GOOS scores, length of stay, reintervention rates, and short- and long-term survival. These findings support SPGJ as a viable and potentially preferable option for managing malignant GOO, although high-quality randomized trials are still needed.

**Supplementary Information:**

The online version contains supplementary material available at 10.1186/s12957-025-04166-6.

## Introduction

Gastric outlet obstruction (GOO) is a clinical syndrome characterized by mechanical blockage at the level of the gastric pylorus or proximal duodenum. In contemporary practice, it is most commonly caused by malignant disease, particularly distal (antral) gastric carcinoma and pancreatic head cancer, but may also result from other periampullary and duodenal tumors, linitis plastica, or metastatic involvement of the upper gastrointestinal tract [1, 2]. Patients typically present with progressive nausea, vomiting, early satiety, abdominal discomfort, and marked nutritional impairment. In advanced gastric cancer, the prevalence of GOO has been reported in approximately 5–15% of cases, and the development of obstruction substantially worsens quality of life and complicates oncologic management [3]. Although malignancy accounts for the majority of GOO in the setting of unresectable upper gastrointestinal cancers, a variety of benign conditions—including peptic ulcer disease, chronic pancreatitis, postoperative strictures, and caustic injury—can also lead to obstruction. Differentiating benign from malignant GOO is essential, as prognosis, therapeutic goals, and optimal management strategies differ substantially between these entities [1–3].

The overarching aim of treatment in malignant GOO is to relieve obstructive symptoms, restore oral intake, and facilitate ongoing systemic therapy. Current practice often favors endoscopic placement of self-expandable metal stents as the initial palliative approach, particularly in patients with limited life expectancy or poor performance status, because stents offer rapid symptom relief with a minimally invasive procedure [4]. Surgical bypass is generally reserved for patients with a longer projected survival, good performance status, or in cases where endoscopic stenting is not feasible or has failed, providing a more durable route for gastric emptying [4].

Conventional gastrojejunostomy (CGJ) is the standard surgical intervention employed for palliation in GOO. This treatment entails establishing an anastomosis between the stomach and jejunum to circumvent the obstruction and reestablish gastrointestinal continuity [5]. CGJ is linked to a significant prevalence of delayed gastric emptying (DGE), affecting as many as 50% of patients [6]. DGE correlates with extended hospitalizations, insufficient oral intake, and postponed chemotherapy treatment, hence diminishing the overall efficacy of palliative care [7, 8].

To mitigate these limitations, stomach-partitioning gastrojejunostomy (SPGJ) was proposed as an alternative to CGJ [7–9]. SPGJ entails the partitioning of the stomach to redirect ingested food straight to the jejunal anastomosis, therefore enhancing gastric emptying and mitigating the risk of DGE [10]. Research has shown the advantages of SPGJ compared to CGJ, especially in decreasing the incidence of DGE, facilitating postoperative oral intake, and improving compliance with palliative chemotherapy [7, 9, 11].

Despite the widespread use of CGJ and the growing interest in SPGJ as a promising alternative, there remains considerable uncertainty regarding the most effective surgery. Although SPGJ has shown potential benefits in reducing DGE and improving postoperative outcomes, most evidence comes from small retrospective studies, single-centre experiences, and limited randomized trials. Given the lack of robust, high-quality evidence directly comparing these two procedures, a comprehensive meta-analysis incorporating both aggregate data and reconstructed individual patient data is essential to provide clearer insights into their relative efficacy and safety. This meta-analysis seeks to evaluate the clinical results of SPGJ and CGJ in patients with unresectable gastric or pancreatic cancers exhibiting GOO.

## Methods

The present investigation adhered to the methodologies described in the Cochrane Handbook of Systematic Reviews on Interventions [12]. The publication was meticulously prepared following the requirements of the Preferred Reporting Items for Systematic Reviews and Meta-Analyses (PRISMA) statement [13]. The study protocol was registered with PROSPERO (CRD420251153331).

### Requirements for eligibility

We refined our research question using the PICO framework as follows:

- Population (P): Adult patients diagnosed with unresectable gastric or pancreatic cancer complicated by gastric outlet obstruction (GOO), deemed suitable for palliative surgical bypass.
- Intervention (I): SPGJ, performed as a palliative surgical intervention for malignant GOO.
- Comparator (C): CGJ, performed for the same palliative indication.
- Outcomes (O): Studies were eligible if they reported at least one of the following clinical outcomes: Incidence of postoperative major complications, Delayed gastric emptying (DGE), Requirement for surgical or endoscopic reintervention, Short-term (30- or 90-day) mortality, Operative time, Gastric Outlet Obstruction Scoring System (GOOS) score, Length of postoperative hospital stay (LOS), Ability to initiate or adhere to postoperative chemotherapy and Overall survival.

#### Study design

Eligible study types included randomized controlled trials (RCTs) and retrospective comparative cohort studies.

#### Exclusion criteria

Review articles, case reports, conference abstracts, non-comparative studies, and animal or preclinical studies were excluded.

### Literature searching and study selection

We performed a comprehensive literature search across four major electronic databases: PubMed, Scopus, Web of Science, the Cochrane Library and ClinicalTrials.gov, including all studies published up to November 25, 2025. The search strategy focused on terms such as stomach-partitioning, Devine procedure, gastric outlet obstruction, and gastrojejunostomy. The detailed search strategies for each database are provided in Supplementary Table 1.

All retrieved citations were imported into EndNote (The EndNote Team, 2025, Clarivate, Philadelphia, PA, USA) for organization and deduplication. Two independent reviewers conducted a two-stage screening process. Initially, titles and abstracts were screened to eliminate irrelevant studies. Subsequently, the remaining full-text articles were assessed for eligibility based on predefined inclusion and exclusion criteria. To ensure comprehensive coverage, the reference lists of included studies and relevant reviews were manually examined to identify additional eligible studies. To minimize selection bias, all stages of study selection and data extraction were conducted using a dual-review process, with disagreements resolved through discussion or by consulting a third reviewer if necessary.

### Data extraction and outcome measures

Two independent reviewers screened titles, abstracts, and full texts to select eligible studies. Disagreements were resolved by a third reviewer. The following data were extracted: Study characteristics: Author, publication year, country, study design, sample size, surgical approach, and follow-up duration. Patient characteristics: Age, sex, body mass index (BMI), primary disease (gastric or pancreatic cancer), and baseline functional status. Clinical outcomes: Postoperative major complications, DGE, need for reintervention, short-term mortality, operation time, GOOS scores, LOS, adherence to postoperative chemotherapy, and overall survival. GOOS, delayed gastric emptying (DGE), and overall survival were prespecified as primary outcomes because they best capture early functional recovery and overall prognosis after palliative bypass. All other endpoints (major complications, length of stay, need for reintervention, and adherence to postoperative chemotherapy) were designated as secondary outcomes and interpreted accordingly.

Whenever available, DGE was defined according to the International Study Group of Pancreatic Surgery (ISGPS) criteria, and major complications were defined as Clavien–Dindo grade III or higher. In studies that did not explicitly apply these classifications, outcomes were extracted as reported by the authors. GOOS was assessed according to the scoring system described in each study, with most studies reporting early postoperative functional status. All outcomes were extracted at the latest timepoints provided in the included reports.

Although quality of life and time to oral intake are clinically meaningful outcomes in malignant GOO, these were inconsistently reported across studies and could not be reliably extracted. Therefore, we focused on DGE, GOOS, and major complications as the most consistently available functional outcomes.

### Reconstruction of individual patient data

To accurately estimate hazard ratios (HRs) for the pooled studies and ensure comprehensive follow-up assessment, we employed an individual patient data (IPD) meta-analysis approach tailored for time-to-event outcomes. This method, based on the two-step approach described by Liu et al. 2021 [14], involves digitizing the time intervals from Kaplan-Meier curves to obtain data on survival probabilities and time points. In the second step, the number at risk at each interval is combined with the extracted survival data to reconstruct individual patient data. We utilized the Shiny application developed by Liu et al. 2021 [14] for data extraction and reconstruction.

### Quality assessment

The quality of the cohort studies was assessed according to the Newcastle-Ottawa quality assessment scale [15] and RCTs were assessed using the Cochrane Risk of Bias 2 (ROB 2) tool [16]. Two authors independently evaluated the quality of the included studies, and in case of any disagreement, the first author took the final decision.

### Statistical analysis

Statistical analyses were conducted using Review Manager (RevMan, version 5.4, The Cochrane Collaboration). For continuous outcomes, mean differences (MD) with 95% confidence intervals (CI) were calculated, while for dichotomous outcomes, relative risks (RR) with 95% CI were reported. For continuous variables reported as medians with interquartile ranges or ranges, we converted these to means and standard deviations using the method of Wan et al. [17], which estimates mean and SD based on sample size and the reported median, IQR, or range. In studies with multi-arm designs, only the relevant intervention and control arms were included to avoid double-counting of participants.

To assess whether study size influenced the effect estimates, we conducted subgroup analyses stratified by sample size (< 45 vs. ≥45 patients). The cut-off of 45 participants was selected because it closely approximated the median sample size of the included studies (46) and provided two well-balanced subgroups, allowing for meaningful comparative analysis. These analyses were conducted for outcomes with sufficient data, including delayed gastric emptying, postoperative major complications, length of hospital stay, and adherence to postoperative chemotherapy. It did not apply to GOOS, the need for reintervention, short-term mortality, and operation time.

Heterogeneity across studies was assessed using the Chi-squared (χ²) test and quantified with the I² statistic, categorized as low (0–25%), moderate (26–50%), or high (> 50%). A random-effects model based on the DerSimonian–Laird estimator was applied for all pooled analyses. Sensitivity analyses were performed using the Hartung–Knapp adjustment and the Paule–Mandel τ² estimator, which demonstrated results consistent with the primary model. For all outcomes, the number of included studies was fewer than 10; therefore, publication bias could not be formally assessed using funnel plots or statistical tests, as recommended by Egger et al. [18].

The certainty of evidence for key outcomes was evaluated using the Grading of Recommendations, Assessment, Development and Evaluation (GRADE) approach. We assessed five domains: risk of bias, inconsistency, indirectness, imprecision, and publication bias. Given that all included studies were observational, the starting level of evidence was set as low and was subsequently upgraded or downgraded based on the presence or absence of methodological strengths and limitations. To strengthen the robustness of the findings, the GRADE assessment was performed, restricted to studies judged at low risk of bias, in accordance with our sensitivity analyses. A “Summary of Findings” table was generated to present the pooled estimates, relative and absolute effects, number of participants, and certainty ratings for the primary outcomes [19].

The reconstructed IPD from Kaplan–Meier curves was analyzed using Jamovi (version 2.5). Cox proportional hazard models were employed to estimate hazard ratios (HRs) with 95% CI. Survival curves were generated to compare CGJ and SPGJ groups and were compared using the log-rank test, with statistical significance set at *p* < 0.05.

## Results

### Literature search

**Fig. 1:**
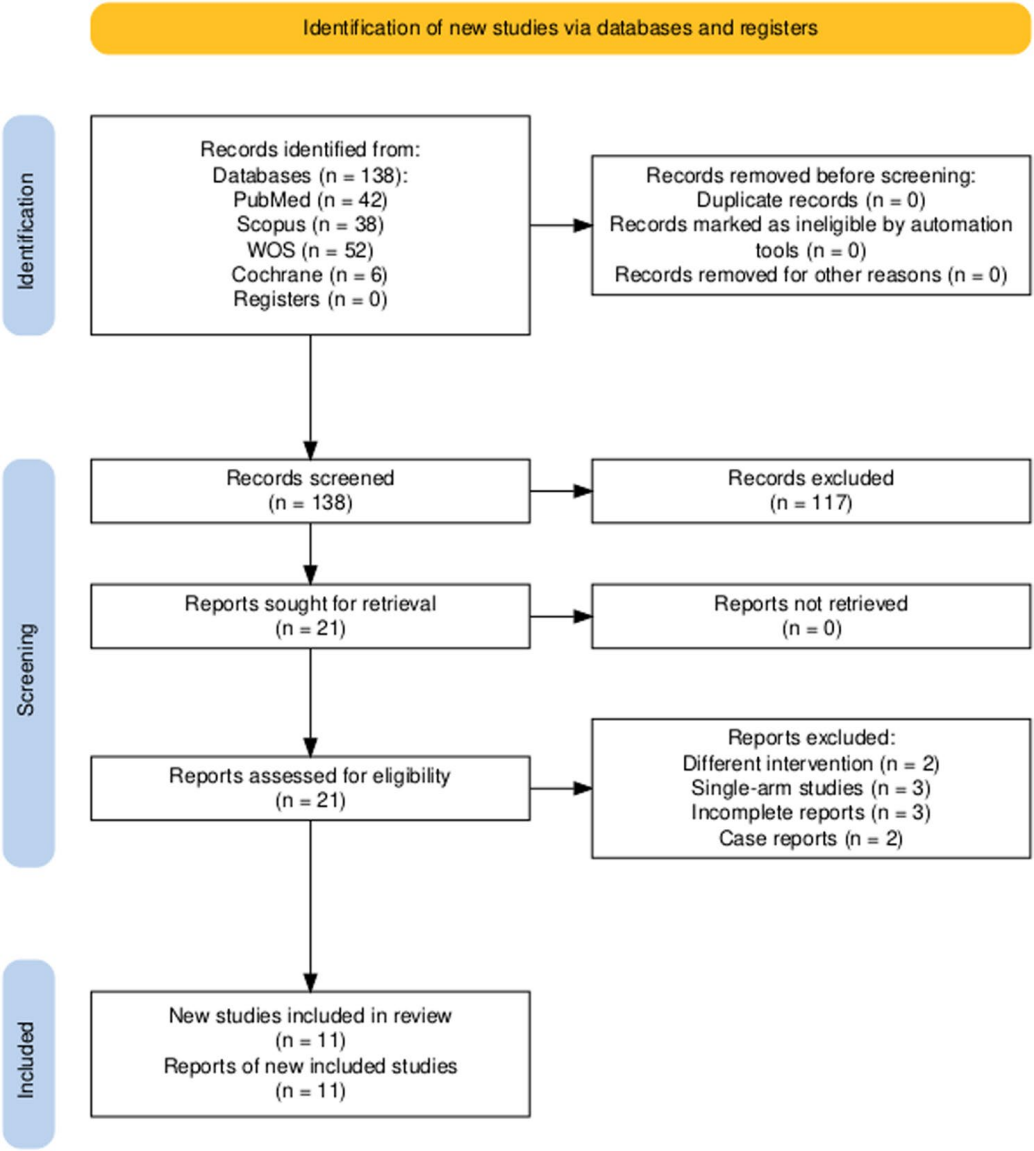
PRISMA flow diagram for study selection

A total of 138 records were identified through database searches, including PubMed (*n* = 42), Scopus (*n* = 38), Web of Science (WOS; *n* = 52), and Cochrane (*n* = 6). No records were obtained from registers. During the screening phase, 117 records were excluded based on title and abstract review. 21 reports were sought for retrieval, and all were successfully retrieved. In the eligibility assessment, 21 full-text reports were assessed. Of these, 10 reports were excluded for the following reasons: different intervention (*n* = 2), single-arm studies (*n* = 3), incomplete reports (*n* = 3), and case reports (*n* = 2). Ultimately, 11 [7, 8, 10, 11, 20–26] were included in the qualitative and quantitative synthesis. Study selection process is illustrated in PRISMA Fig. 1 and PRISMA-2020 checklist (Supplementary File 2).

### Characteristics of the included studies

The systematic review included 11 studies published between 1997 and 2025 across diverse geographic regions including Asia, Europe, and South America. There were 10 retrospective studies [8, 10, 11, 20–26] and one RCT [7]. The cumulative sample comprised 456 patients with gastric outlet obstruction (GOO), predominantly due to malignancies (gastric cancer: 54.6%, pancreatic/biliary cancer: 23.2%, other malignancies: 5.9%, and benign conditions: 16.3%). Sample sizes ranged from 10 to 73 patients across studies (Table 1).

**Table 1:** Characteristics of the included studies and comparative outcomes of surgical approaches for gastric outlet obstruction note: this table summarizes key study characteristics

Study	Study design	Year	Country	Study Period	Sample Size	Patient Diagnoses	Surgical Approach	Follow-up	Main Outcomes
Abdel-lah-Fernändez 2015	Retrospective	2015	Spain	12 years	22	Unresectable distal gastric cancer	(Group I), conventional gastrojejunostomy (Group II), and stomach-partitioning gastrojejunostomy (Group III)	NR	SPGJ allowed normal diet at 15th day (*p* < 0.05) Lower morbidity in SPGJ (42% vs. 66.7%) No reinterventions in SPGJ group Superior median survival in SPGJ (6.5 months)
Dolan 2003	Retrospective	2003	United Kingdom	1993–1997	10	Unresectable distal gastric cancer with pancreatic invasion	Open approach	3–4 months median	No mortality in either group Exclusion: free fluids by day 5, diet by day 7 Shorter hospital stays (11 days both groups) + I13 Median survival: 3 mo (CGJ) vs. 4 mo (exclusion)
Ernberg 2015	Retrospective	2015	Sweden	NR	24	GOO from various causes, primarily pancreatic cancer	Both laparoscopic and open	50 ± 30 days	DGE grade B-C: 0% in PSPGJ vs. 42.9% in CGJ (*p* = 0.024) Oral nutrition only: 100% in PSPGJ vs. 30.8% in CGJ (*p* = 0.002) Shorter hospital stay in PSPGJ (11.5 vs. 22.5 days, *p* = 0.023)
Hai 2024	Retrospective	2023	Vietnam	2018–2022	52 (after PSM)	Unresectable gastric cancer with GOO	Mix: laparoscopic and open	SPGJ: 4.6 months > CGJ: 3.6 months	Lower DGE in SPGJ (3.8% vs. 34.6%, *p* = 0.013) Less vomiting (3.8% vs. 42.3%, *p* = 0.002) Shorter time to solid diet (4.1 vs. 5.7 days, *p* = 0.021) Shorter hospital stay (7.7 vs. 9.3 days, *p* = 0.014)
Huang 2022	Retrospective	2022	Sweden	2013–2020	43	Mixed: gastric cancer (50%), pancreatic/ampullary (28%), other malignancy (9%), benign (13%)	PSPGJ: 59% laparoscopic CGJ: 45% laparoscopic	NR	PSPGJ improved GOOSS by 0.63 points (*p* = 0.041) No procedure-related complications GOO recurrence: 19% in PSPGJ Similar GOOSS improvement between group Lower complication rate in PSPGJ
Kaminishi 1997	Retrospective	1997	Japan	NR	21 (8 SPGJ, 13 GJ)	Unresectable gastric carcinoma (stage IV)	SPGJ vs. conventional GJ	Not specified, reports 1-year survival	Regular meal at 2 weeks: 88% SPGJ vs. 31% GJ (*p* < 0.05) − 1-year survival: 42.9% SPGJ vs. 7.7% GJ (*p* < 0.05) - Mean survival: 13.4 months vs. 5.8 months (*p* < 0.05)
Oida 2009	Retrospective	2009	Japan	NR	60	Unresectable advanced gastric and pancreatic cancer	MDVSR* (*n* = 30) vs. CGJ (*n* = 30)	NR	Faster resumption of solid food with MDVSR - Lower DGE in MDVSR (6.7% vs. 46.7%, *p* < 0.009) - Shorter hospital stay (11 vs. 17 days, *p* < 0.00028) - Longer survival with MDVSR (192 vs. 103 days, *p* < 0.0019)
Ramos 2025	Randomized-controlled trial	2025	Brazil	2013–2020	52	Obstructive unresectable distal gastric cancer	SPGJ (*n* = 27) vs. CGJ (*n* = 25)	NR	Higher rate of GOOS 3 in SPGJ (96% vs. 72%, *p* = 0.037) - Similar complications and mortality - Higher RBC transfusions in SPGJ group - Non-significant survival difference (12.4 vs. 5.3 months, *p* = 0.154)
Usuba 2010	Retrospective	2011	Japan	Jan 2000-Dec 2007	46 (26 MDE, 20 CGJ)	Unresectable pancreaticobiliary cancer	MDE vs. CGJ	Until postoperative survival (mean ~ 102–152 days)	DGE: 23% MDE vs. 40% CGJ No oral intake: 0% MDE vs. 10% CGJ Hospital stay: ~30 days MDE vs. ~ 32 days CGJ MDE effective for duodenal bleeding cases
Yildirim 2020	Retrospective	2020	Turkey	Mar 2013-Feb 2019	53 (16 SPGJ, 37 CGJ)	Malignant gastric outlet obstruction	SPGJ vs. CGJ	30-day postoperative	DGE: 18.8% SPGJ vs. 70.3% CGJ Clinically significant DGE: 0% SPGJ vs. 27% CGJ Better oral intake in SPGJ group Major complications: 6.3% SPGJ vs. 24.3% CGJ
Zhang 2023	Retrospective	2023	China	Jan 2015-Mar 2022	73 (48 SPGJ, 25 CGJ)	GOO (gastric cancer or benign pyloric obstruction)	SPGJ vs. CGJ	30-day postoperative	DGE: 2.1% SPGJ vs. 36% CGJ Time to oral intake: 3 vs. 4 days (*p* = 0.001) Hospital stay: 7 vs. 9 days (*p* < 0.001) Fluid velocity: 29 mm/s SPGJ vs. 22 mm/s CGJ

Note: This table summarizes key study characteristics. Abbreviations: *GOO* Gastric Outlet Obstruction, *SPGJ* Stomach-Partitioning Gastrojejunostomy, *CGJ* Conventional Gastrojejunostomy, *PSPGJ* Pylorus-Sparing Gastrojejunostomy, *MDVSR* Modified Devine’s Subserosal Resection, *MDE* Modified Double-Bypass Enterostomy, *DGE* Delayed Gastric Emptying, *GOOS* Gastric Outlet Obstruction Scoring System, *NR* Not Reported

Patients were predominantly male (67.3% vs. 32.7% female) with a mean age ranging from 62 to 77 years. The mean BMI across studies reporting this measure was 21.3 ± 3.0 kg/m². Where reported, Eastern Cooperative Oncology Group (ECOG) performance status was primarily 0–1, indicating good functional status. Local disease was present in 38.2% of patients while 61.8% had metastatic disease. Most procedures (72.6%) were performed via open approach, with 27.4% completed laparoscopically.

The primary comparison was between SPGJ variants (*n* = 216) and CGJ (*n* = 240). Variations of SPGJ included pylorus-sparing gastrojejunostomy (PSPGJ), modified Devine’s subserosal resection (MDVSR), and modified double-bypass enterostomy (MDE). Roux-en-Y reconstruction was utilized in 85.2% of SPGJ procedures and 64.7% of CGJ procedures (Table 2).

**Table 2:** Baseline patient characteristics and surgical details in included studies

Study	Group	Patients	Age (years)	Sex (M: F)	BMI (kg/m²)	ECOG Status (0/1/2+)	Primary Diagnosis (Gastric/Pancreatic/Other/Benign)	Tumor Stage (Local/Metastatic)	Surgical Approach (Open/Lap/Converted)	Reconstruction (Roux-en-Y/Other)	Chemotherapy (Pre/Post)
Abdel-lah-Fernändez 2015	SPGJ	7	77	5:2 (71:29%)	-	-	7/0/0/0 (100%/0%/0%/0%)	1/6 (14/86%)	7/0/0 (100%/0%/0%)	7/0 (100%/0%)	-
	CGJ	9	75	7:2 (78:22%)	-	-	9/0/0/0 (100%/0%/0%/0%)	4/5 (56/44%)	9/0/0 (100%/0%/0%)	-	-
Dolan 2003	SPGJ	6	73 (66–82)	5:1 (83:17%)	-	-	-	-	-	-	-
	CGJ	4	73 (61–78)	3:1 (75:25%)	-	-	-	-	-	-	-
Ernberg 2015	SPGJ	10	71.5 (24–83)	7:3 (70:30%)	-	-	6/1/1/2 (60/10/10/20%)	2/6 (25/75%)	4/6/- (40/60/-)	10/0 (100/0%)	-
	CGJ	14	64.5 (21–83)	8:6 (57:43%)	-	-	1/6/6/1 (7/43/43/7%)	3/9 (25/75%)	9/5/- (64/36/-)	3/11 (21/79%)	-
Hai 2024	SPGJ	26	64.5 ± 14.4	17:9 (65:35%)	18.6 ± 3.8	13/13/0 (50/50/0%)	16/5/1/4 (62/19/4/15%)	5/21 (19/81%)	21/5/0 (81/19/0%)	26/0 (100/0%)	3/12 (12/46%)
	CGJ	26	65.2 ± 15.2	19:7 (73:27%)	19.5 ± 3.3	12/14/0 (46/54/0%)	5/15/6/0 (19/58/23/0%)	7/19 (27/73%)	21/5/0 (81/19/0%)	26/0 (100/0%)	2/12 (8/46%)
Huang 2022	SPGJ	32	64 (23–82)	19:13 (59:41%)	22 ± 3.1	15/16/1 (47/50/3%)	16/9/3/4 (50/28/9/13%)	-	10/19/3 (31/59/10%)	31/1 (97/3%)	7/- (22/-)
	CGJ	11	67 (41–82)	4:7 (36:64%)	23 ± 3.6	2/6/3 (18/55/27%)	5/5/0/1 (45/45/0/10%)	-	6/5/0 (55/45/0%)	11/0 (100/0%)	0/- (0/-)
Kaminishi 1997	SPGJ	8	64 (47–69)	8:0 (100:0%)	-	-	7/0/1/0 (88/0/12/0%)	All stage IV	-	-	-/7 (-/88%)
	CGJ	13	62 (50–80)	9:4 (69:31%)	-	-	13/0/0/0 (100/0/0/0%)	All stage IV	-	-	-/10 (-/77%)
Oida 2009	SPGJ	30	71 ± 8	21/9 (70%/30%)	-	-	24/6/0/0 (80%/20%/0%/0%)	3/27 (10%/90%)	30/0/0 (100%/0%/0%)	-	-
	CGJ	30	69 ± 8	22/8 (73%/27%)	-	-	23/7/0/0 (77%/23%/0%/0%)	3/27 (10%/90%)	30/0/0 (100%/0%/0%)	-	-
Ramos 2025	SPGJ	27	62.7 ± 10.2	17/10 (63%/37%)	21.3 ± 3.4	0/15/12 (0%/55.6%/44.4%)	13/3/0/0 (81.2%/18.8%/0%/0%)	10/6 (62.5%/37.5%)	25/2/0 (93%/7%/0%)	27/0 (100%/0%)	3/22 (18.8%/81.5%)
	CGJ	25	68.7 ± 14.4	21/4 (84%/16%)	20.7 ± 4.0	0/18/7 (0%/72%/28%)	20/17/0/0 (54.1%/45.9%/0%/0%)	25/12 (67.6%/32.4%)	25/0/0 (100%/0%/0%)	21/4 (84%/16%)	4/15 (10.8%/60%)
Usuba 2010	SPGJ	26	64.2 ± 11.0	17/9 (65%/35%)	-	-	0/20/6/0 (0%/77%/23%/0%)	10/16 (38%/62%)	26/0/0 (100%/0%/0%)	-	4/10 (15%/38%)
	CGJ	20	63.9 ± 12.0	12/8 (60%/40%)	-	-	0/13/7/0 (0%/65%/35%/0%)	6/14 (30%/70%)	20/0/0 (100%/0%/0%)	-	7/10 (35%/50%)
Yildirim 2020	SPGJ	16	62.7 ± 10.2	11/5 (68.8%/31.2%)	21.3 ± 3.4	-	13/3/0/0 (81.2%/18.8%/0%/0%)	10/6 (62.5%/37.5%)	14/2/0 (87.5%/12.5%/0%)	16/0 (100%/0%)	3/ - (18.8%/ -)
	CGJ	37	68.7 ± 14.4	25/12 (67.6%/32.4%)	20.7 ± 4.0	-	20/17/0/0 (54.1%/45.9%/0%/0%)	25/12 (67.6%/32.4%)	37/0/0 (100%/0%/0%)	21/16 (56.8%/43.2%)	4/ - (10.8%/ -)
Zhang 2023	SPGJ	48	63.9 ± 10.3	36/12 (75%/25%)	21.8 ± 2.7	-	38/0/0/10 (79.2%/0%/0%/20.8%)	-	6/42/0 (12.5%/87.5%/0%)	-	10/ - (38%/ -)
	CGJ	25	65.2 ± 11.0	14/11 (56%/44%)	21.4 ± 3.1	-	20/0/0/5 (80%/0%/0%/20%)	-	7/18/0 (28%/72%/0%)	-	10/ - (50%/ -)

Notes: Patient demographics and clinical characteristics across the included studies demonstrate comparable baseline status between intervention groups. Abbreviations: *SPGJ* Stomach-Partitioning Gastrojejunostomy, *PSPGJ* Partial Stomach-Partitioning Gastrojejunostomy, *CGJ* Conventional Gastrojejunostomy, *PGE* Partial Gastric Exclusion, *Lap* Laparoscopic, *BMI* Body Mass Index, *ECOG* Eastern Cooperative Oncology Group performance status, *M* Male, *F* Female

### Quality of the included studies

**Fig. 2:**
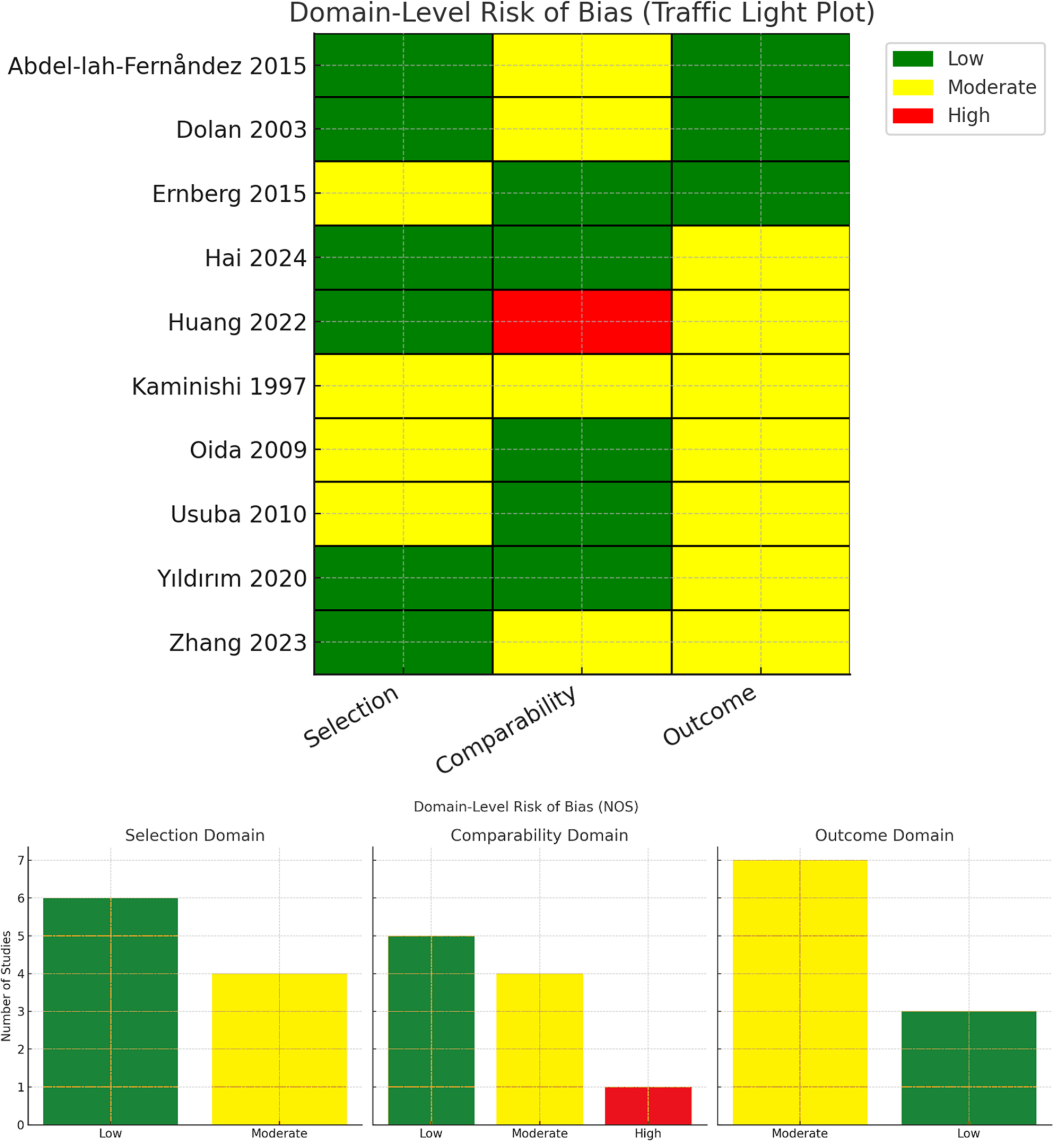
Risk of bias assessment for the included observational studies

**Fig. 3:**
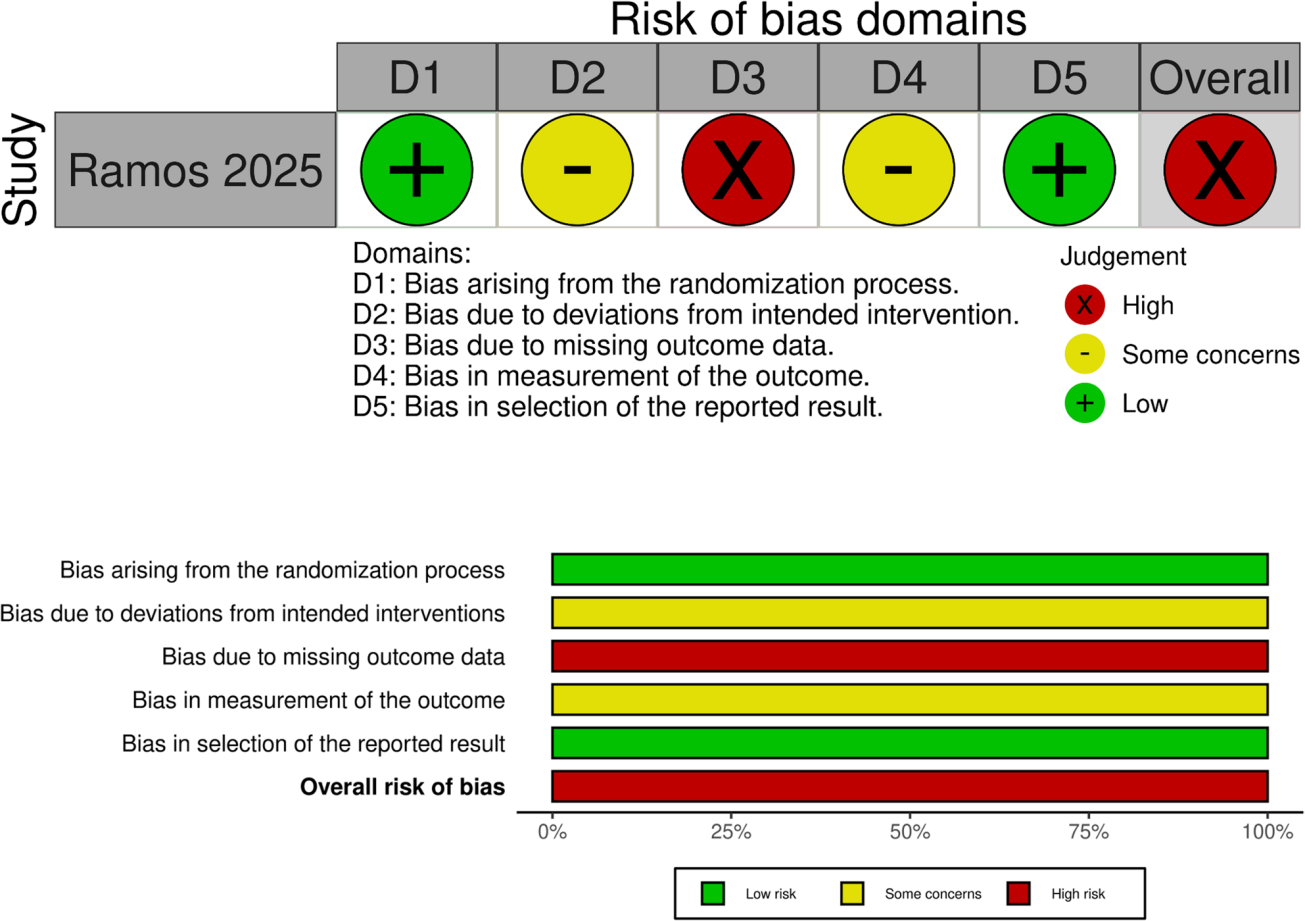
Risk of bias assessment for the included randomized-controlled trials

Using the Newcastle-Ottawa Scale, six studies demonstrated low risk of bias (scores 7–8) while four had moderate risk (scores 5–6). Common limitations included non-representative cohort selection and inadequate follow-up duration (Fig. 2). Ramos et al. 2025 [7] study was evaluated using the ROB2 tool, showed low risk in the randomization process and selective reporting domains, but was rated as high risk overall due to significant post-randomization exclusions (42%) and concerns related to its open-label design affecting outcome measurement and intervention adherence; Fig. 3.

### Primary clinical outcomes

#### Gastric outlet obstruction scoring system

**Fig. 4:**
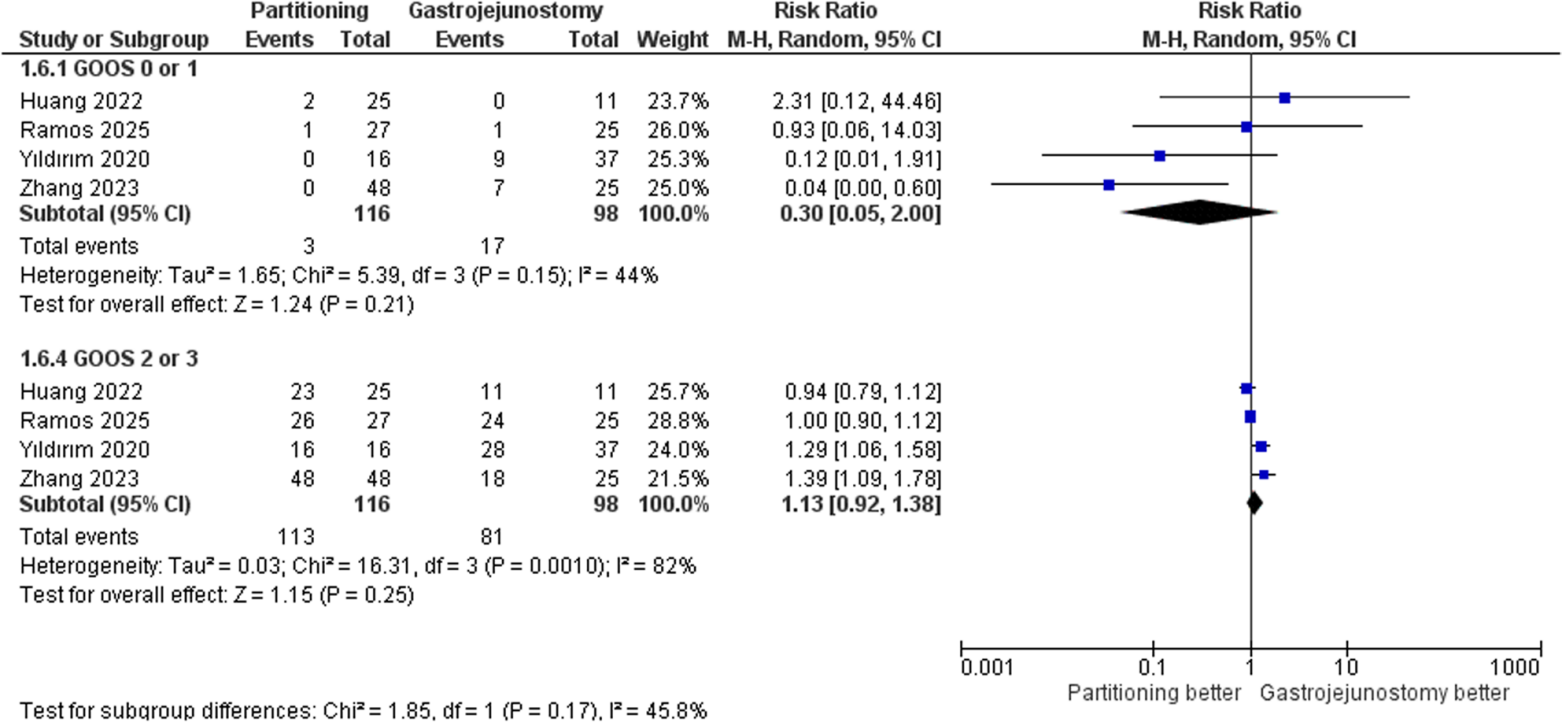
Forest plot of gastric outlet obstruction scoring system (GOOS)

For the GOOS 0 or 1 (poor functional status), the pooled analysis was not statistically significant (RR = 0.30, 95% CI: 0.05 to 2.00, *p* = 0.21). Moderate heterogeneity was present (τ² = 1.65; I² = 44%), this heterogeneity was not resolved in the leave-one-out test. For the GOOS 2 or 3 (good functional status), there was no significant difference between the two groups (RR = 1.13, 95% CI: 0.92 to 1.38, *p* = 0.25). Substantial heterogeneity was observed (τ² = 0.03; I² = 82%), this heterogeneity was not resolved in the leave-one-out test; Fig. 4.

#### Delayed gastric emptying

**Fig. 5:**
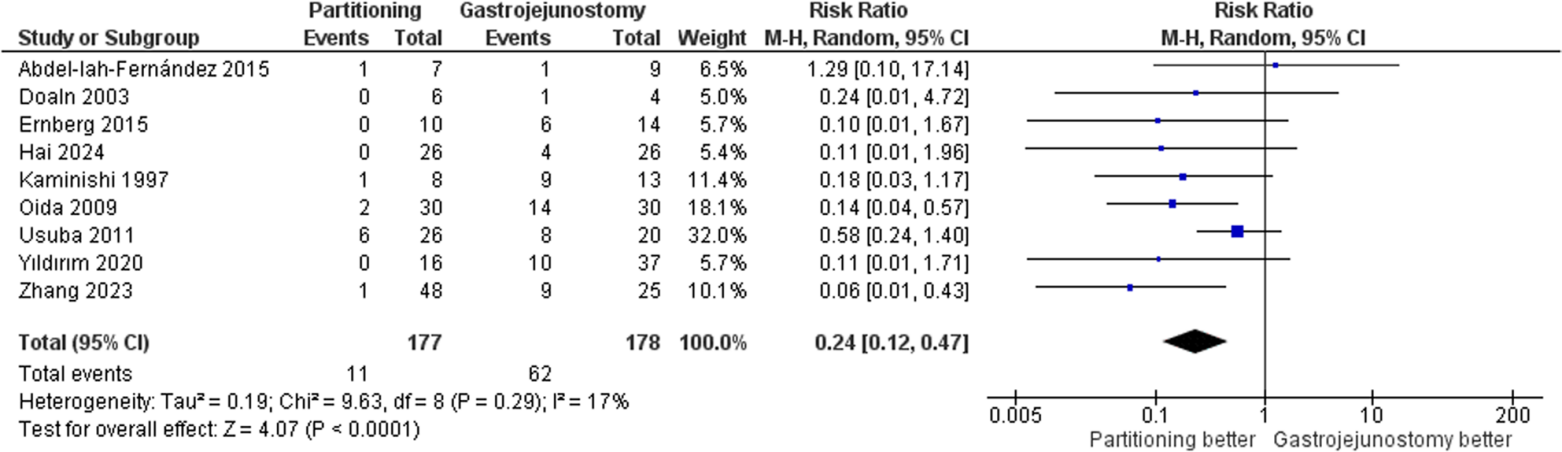
Forest plot of delayed gastric emptying (DGE)

The pooled analysis demonstrated a significantly lower risk of DGE in the SPGJ group compared to the CGJ group (RR = 0.24, 95% CI: 0.12 to 0.47, *p* < 0.0001); Fig. 5. Statistical heterogeneity was low (τ² = 0.19; I² = 17%). After excluding Zhang et al. [25] in a leave-one-out test, the heterogeneity was resolved (I² = 0%), showing a similar effect size (RR = 0.31, 95% CI: 0.17 to 0.57, *p* = 0.0002); Supplementary Fig. 1. There was no statistically significant difference between subgroups (*p* = 0.72) and (I² = 0%). Supplementary Fig. 2.

#### Reconstructed survival analysis

**Fig. 6:**
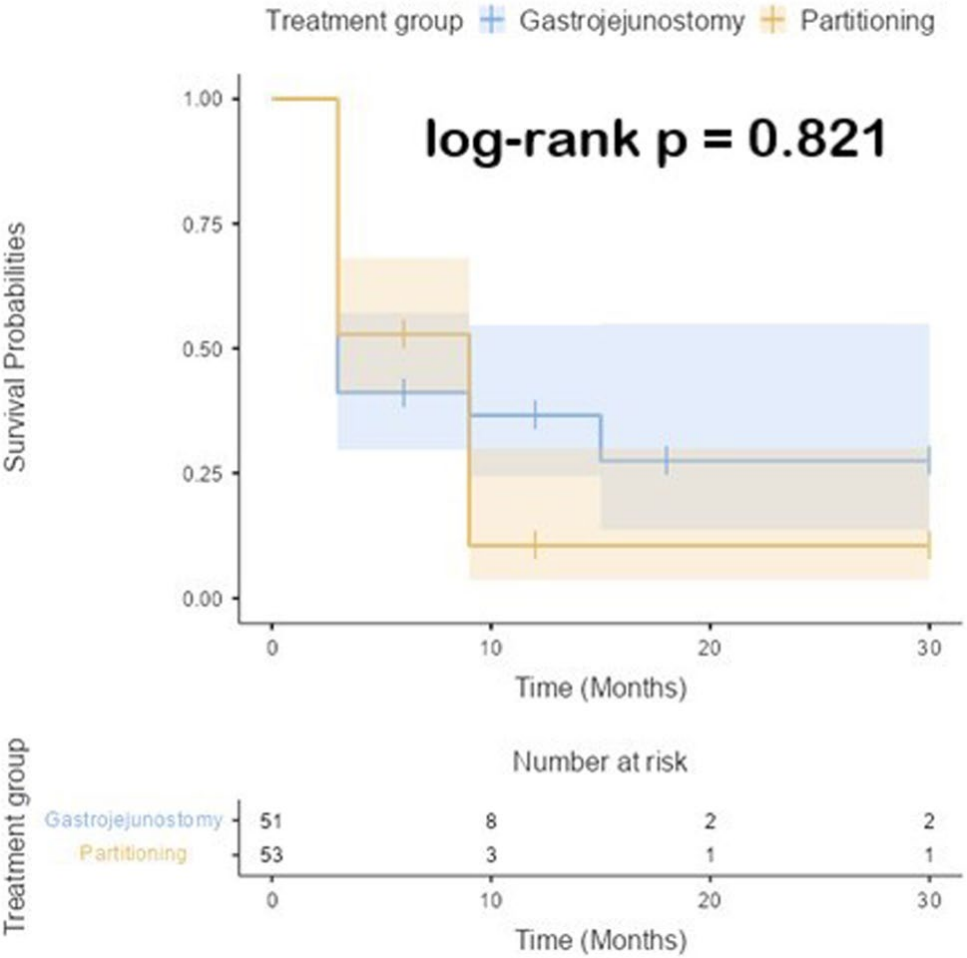
Kaplan-Meier curve of survival

A reconstructed IPD K-M survival analysis was performed to compare the survival of SPGJ and CGJ. The median survival time for patients in the CGJ group was 3 months (95% CI: 3 to NA), while for the SPGJ group, the median survival time was 9 months (95% CI: 3 to 9). The log-rank test demonstrated no statistically significant difference in survival between the two groups (*p* = 0.821); Fig. 6. Consistently, the Cox regression analysis revealed a HR of 1.06 for SPGJ relative to CGJ (95% CI: 0.66 to 1.70, *p* = 0.822), indicating no significant difference in the risk of mortality between the two procedures. Although the median survival times differed numerically, this result should be interpreted with caution, as the confidence intervals were wide and overlapping. The survival curves demonstrated substantial overlap, and both the log-rank test and Cox model indicated no statistically significant difference across the follow-up period. The estimated survival rates at 6 months, 12 months, 24 months, and 30 months for both groups are shown in Table 3.

**Table 3:** Survival probability for each timepoint for the study arms

Timepoint (Months)	Treatment groups	Survival probability	Lower 95% CI	Upper 95% CI
0	Gastrojejunostomy	100.0%	100.0%	100.0%
	Partitioning	100.0%	100.0%	100.0%
6	Gastrojejunostomy	41.2%	29.7%	57.2%
	Partitioning	52.8%	41.0%	68.1%
12	Gastrojejunostomy	36.6%	24.5%	54.7%
	Partitioning	10.6%	3.7%	30.0%
24	Gastrojejunostomy	27.5%	13.7%	54.9%
	Partitioning	10.6%	3.7%	30.0%
30	Gastrojejunostomy	27.5%	13.7%	54.9%
	Partitioning	10.6%	3.7%	30.0%

### Secondary clinical outcomes

#### Postoperative major complications

**Fig. 7:**
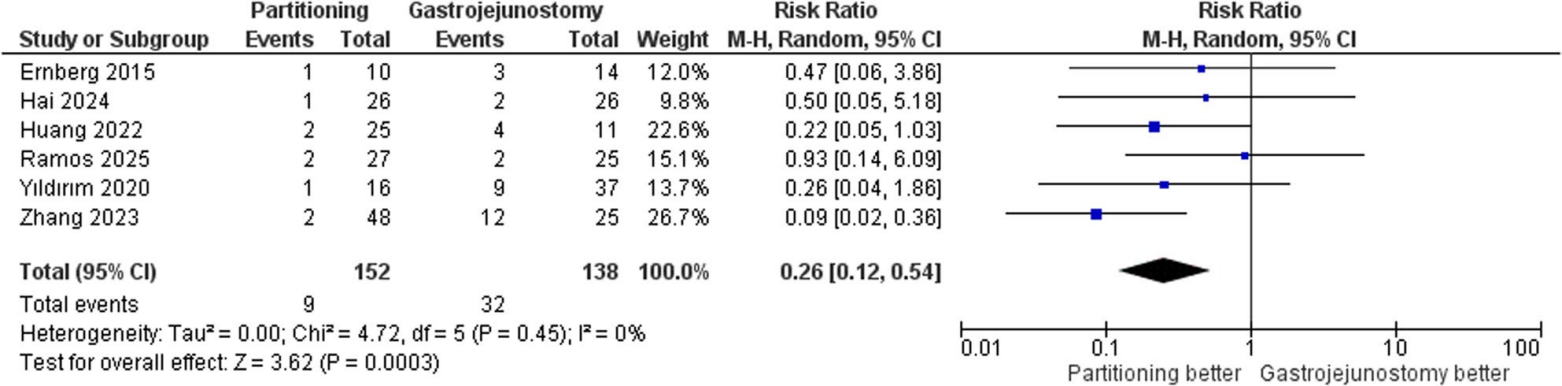
Forest plot of major complications

SPGJ was associated with a significantly lower risk of postoperative major complications compared to CGJ (RR = 0.26, 95% CI: 0.12–0.54, *p* = 0.0003); Fig. 7. Heterogeneity was low (τ² = 0.00; I² = 0%), indicating consistency across studies. There was no statistically significant difference between subgroups (*p* = 0.95) and (I² = 0%). Supplementary Fig. 3.

#### Need for reintervention

**Fig. 8:**
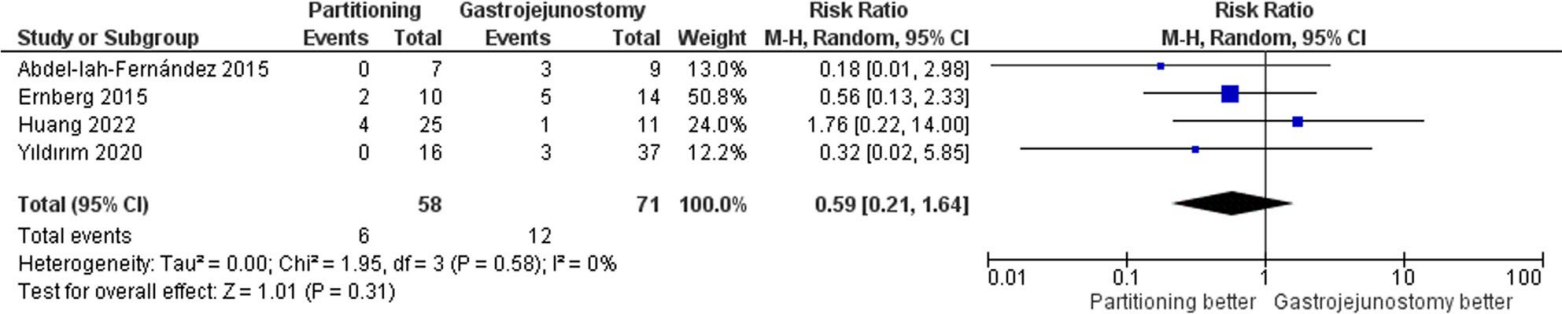
Forest plot of need for reintervention

The pooled analysis showed no significant difference between SPGJ and CGJ in the need for postoperative reintervention (RR = 0.59, 95% CI: 0.21–1.64, *p* = 0.31); Fig. 8. There was no heterogeneity among the studies (τ² = 0.00; I² = 0%).

#### Short-term mortality

**Fig. 9:**
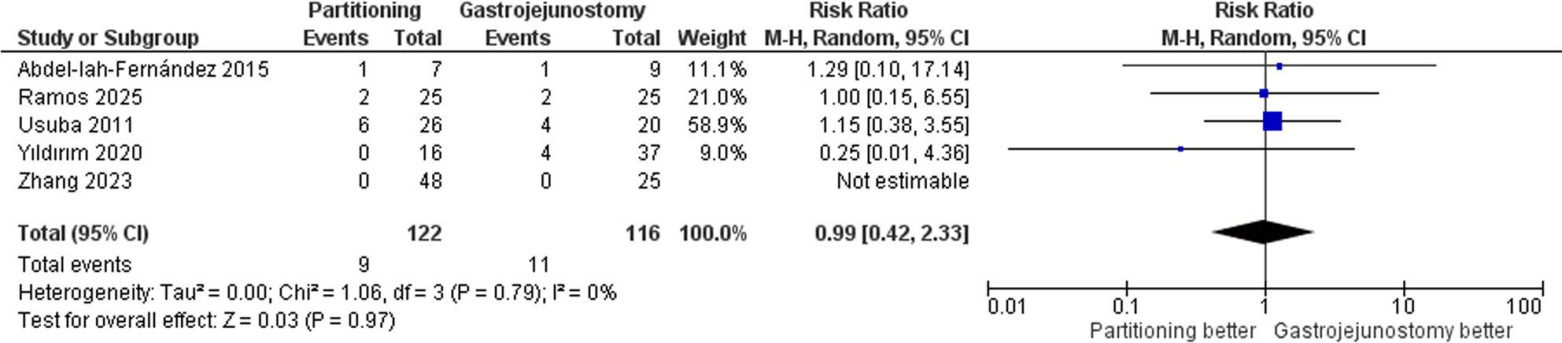
Forest plot of short-term mortality

The pooled risk ratio was 0.99 (95% CI: 0.42–2.33, *p* = 0.97), indicating no significant difference between SPGJ and CGJ in short-term mortality; Fig. 9. No heterogeneity was detected (τ² = 0.00; I² = 0%).

#### Operation time

**Fig. 10:**
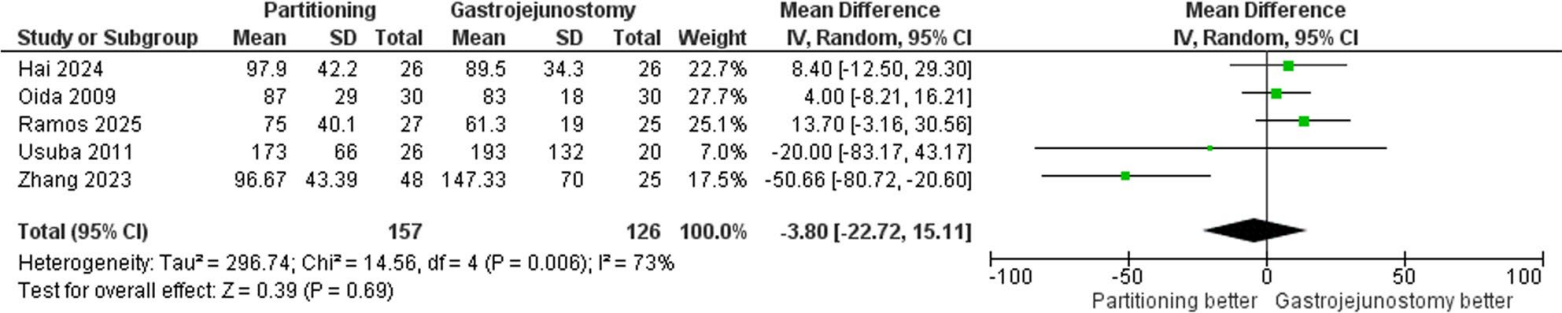
Forest plot of operation time

The meta-analysis of operation time showed no significant difference between SPGJ and CGJ, MD = -3.80 min (95% CI: -22.72, 15.11; *p* = 0.69); Fig. 10. Heterogeneity among the included studies was substantial **(**τ² = 296.74; I² = 73%**).** After excluding Zhang et al. [25], heterogeneity was effectively eliminated (I² = 0%), with a MD of 6.99 min (95% CI: -1.86, 15.84; *p* = 0.12), suggesting no statistically significant difference between the two surgical techniques; Supplementary Fig. 4.

#### Length of hospital stay

**Fig. 11:**
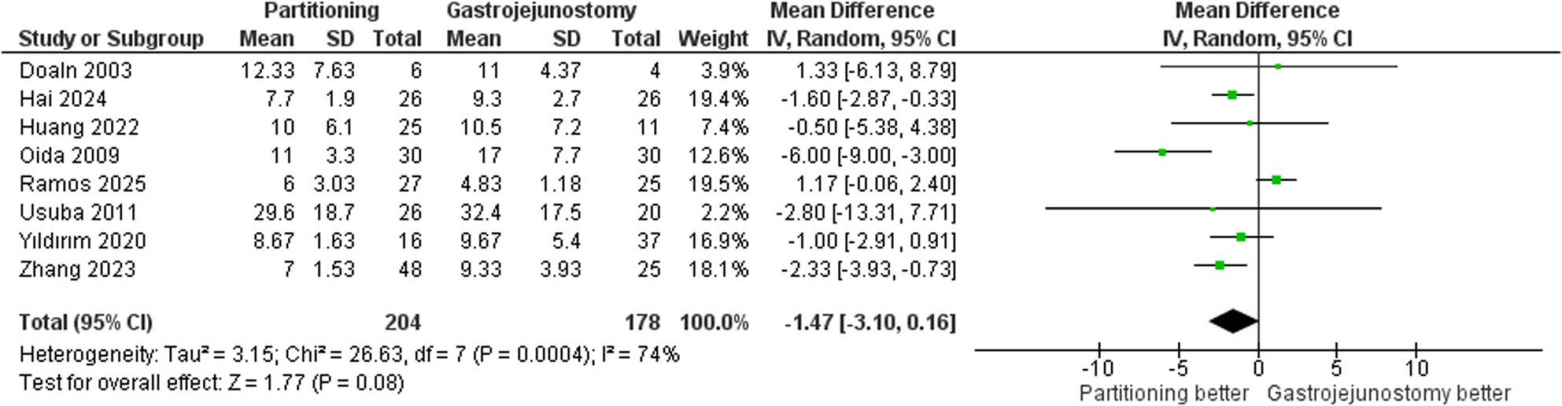
Forest plot of length of hospital stay (LOS)

The meta-analysis demonstrated no statistically significant difference (MD = -1.47, 95% CI: -3.10 to 0.16, *p* = 0.08); Fig. 11. Considerable heterogeneity was detected (τ² = 3.15; I² = 74%), this heterogeneity was not resolved in the leave-one-out test. There was no statistically significant difference between subgroups (*p* = 0.95) and (I² = 0%). Supplementary Fig. 5.

#### Adherence to postoperative chemotherapy

**Fig. 12:**
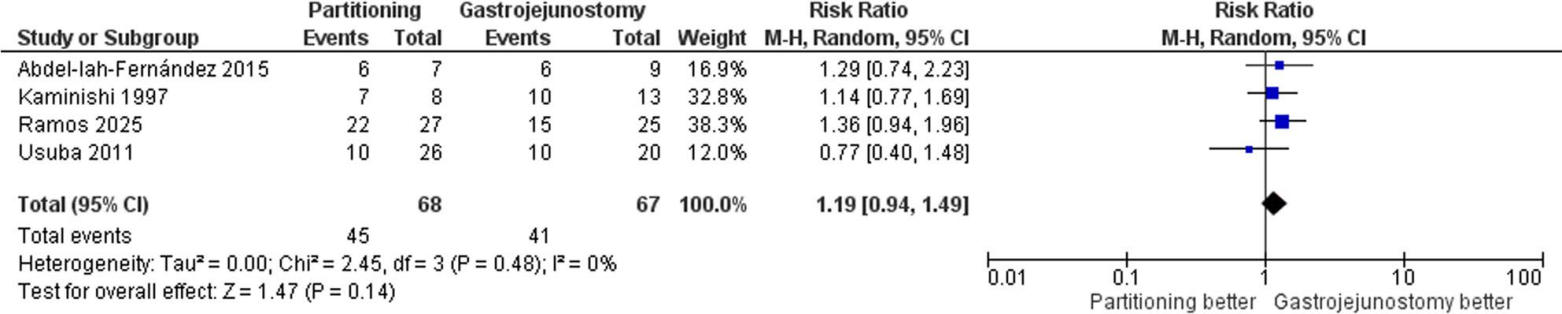
Forest plot of adherence to postoperative chemotherapy

The pooled analysis revealed no significant difference between the groups regarding adherence to postoperative chemotherapy (RR = 1.19, 95% CI: 0.94 to 1.49, *p* = 0.14); Fig. 12. No evidence of heterogeneity was observed (τ² = 0.00; I² = 0%). There was no statistically significant difference between subgroups (*p* = 0.95) and (I² = 0%). Supplementary Fig. 6.

### Sensitivity analyses restricted to low-bias studies for primary outcomes

For the GOOS 0 or 1 (poor functional status), the pooled analysis was statistically significant (RR = 0.07, 95% CI: 0.01 to 0.47, *p* = 0.007). Heterogeneity was not present (I² = 0%). For the GOOS 2 or 3 (good functional status), there was a significant difference between the two groups (RR = 1.33, 95% CI: 1.14 to 1.56, *p* = 0.0003). Heterogeneity was not observed (I² = 0%). Supplementary Fig. 7.

The pooled analysis demonstrated a significantly lower risk of DGE in the SPGJ group compared to the CGJ group (RR = 0.16, 95% CI: 0.05 to 0.50, *p* = 0.002); statistical heterogeneity was not present (I² = 0%). Supplementary Fig. 8.

### GRADE approach restricted to low-bias studies for primary outcomes

The GRADE assessment demonstrated that SPGJ, compared with CGJ, was associated with clinically meaningful improvements in functional outcomes. Patients undergoing partitioning had a markedly reduced risk of poor oral intake (GOOS 0–1), with moderate certainty of evidence. Conversely, the likelihood of achieving adequate oral intake (GOOS 2–3) was significantly higher in the partitioning group, supported by high certainty of evidence. In addition, the risk of delayed gastric emptying was substantially lower with partitioning, also supported by moderate certainty of evidence. These findings collectively suggest that SPGJ confers important clinical advantages over CGJ in the management of malignant gastric outlet obstruction. (Tables 4 and 5).

**Table 4:** GRADE evidence profile

Certainty assessment							№ of patients		Effect		Certainty	Importance
№ of studies	Study design	Risk of bias	Inconsistency	Indirectness	Imprecision	Other considerations	Partitioning	Gastrojejunostomy	Relative (95% CI)	Absolute (95% CI)		
GOOS 0 or 1												
2	non-randomised studies	not serious	not serious	not serious	not serious	strong association	0/64 (0.0%)	16/62 (25.8%)	**RR 0.07** (0.01 to 0.47)	**240 fewer per 1**,**000** (from 255 fewer to 137 fewer)	⨁⨁⨁◯ Moderate	CRITICAL
**GOOS 2 or 3**												
2	non-randomised studies	not serious	not serious	not serious	not serious	very strong association	64/64 (100.0%)	46/62 (74.2%)	**RR 1.33** (1.14 to 1.56)	**245 more per 1**,**000** (from 104 more to 415 more)	⨁⨁⨁⨁ High	CRITICAL
**DGE**												
5	non-randomised studies	not serious	not serious	not serious	not serious	strong association	2/87 (2.3%)	27/89 (30.3%)	**RR 0.16** (0.05 to 0.50)	**255 fewer per 1**,**000** (from 288 fewer to 152 fewer)	⨁⨁⨁◯ Moderate	CRITICAL

*CI* confidence interval, *RR* risk ratio

**Table 5:** Summary of findings table

Partitioning compared to Gastrojejunostomy for gastric outlet obstruction						
Outcomes	Anticipated absolute effects^*^ (95% CI)		Relative effect (95% CI)	№ of participants (studies)	Certainty of the evidence (GRADE)	Comments
	**Risk with Gastrojejunostomy**	**Risk with Partitioning**				
GOOS 0 or 1	258 per 1,000	**18 per 1**,**000** (3 to 121)	**RR 0.07** (0.01 to 0.47)	126 (2 non-randomised studies)	⨁⨁⨁◯ Moderate	Partitioning probably results in a reduction in GOOS 0 or 1 .
GOOS 2 or 3	742 per 1,000	**987 per 1**,**000** (846 to 1,000)	**RR 1.33** (1.14 to 1.56)	126 (2 non-randomised studies)	⨁⨁⨁⨁ High	Partitioning results in large increase in GOOS 2 or 3.
DGE	303 per 1,000	**49 per 1**,**000** (15 to 152)	**RR 0.16** (0.05 to 0.50)	176 (5 non-randomised studies)	⨁⨁⨁◯ Moderate	Partitioning probably results in a reduction in DGE .

*The risk in the intervention group (and its 95% confidence interval) is based on the assumed risk in the comparison group and the relative effect of the intervention (and its 95% CI).CI: confidence interval; RR: risk ratio

GRADE Working Group grades of evidence

High certainty: we are very confident that the true effect lies close to that of the estimate of the effect

Moderate certainty: we are moderately confident in the effect estimate: the true effect is likely to be close to the estimate of the effect, but there is a possibility that it is substantially different

Low certainty: our confidence in the effect estimate is limited: the true effect may be substantially different from the estimate of the effect

Very low certainty: we have very little confidence in the effect estimate: the true effect is likely to be substantially different from the estimate of effect

## Discussion

Our meta-analysis indicates that, compared with CGJ, SPGJ provides consistent early postoperative advantages but does not clearly improve downstream outcomes. SPGJ was associated with a markedly lower risk of delayed gastric emptying and fewer major postoperative complications, while functional status (GOOS), reintervention rates, length of hospital stay, adherence to postoperative chemotherapy, short-term mortality, and overall survival were broadly similar between the two techniques. These findings suggest that the benefits of SPGJ are largely confined to the early physiological phase of recovery rather than translating into demonstrable improvements in global functional recovery, oncologic treatment continuity, or survival.

Our results showed no significant difference between SPGJ and CGJ in achieving GOOS scores of 0–1 or 2–3. Our results align with the results of studies by Ramos et al., Huang et al., and Yildirim [7, 22, 26]. This finding suggests that while SPGJ may effectively reduce DGE, it does not necessarily result in superior GOOS scores compared to CGJ. This discrepancy could be due to GOOS primarily reflecting oral intake ability, which may be influenced by overall nutritional status and patient condition rather than the specific surgical technique. Although GOOS provides valuable short-term outcome data during hospitalization, it does not address long-term outcomes like chemotherapy adherence and overall survival.

Our meta-analysis demonstrated a significant reduction in the incidence of DGE following SPGJ compared to CGJ. This finding highlights a substantial clinical benefit of SPGJ in minimizing postoperative DGE. The results align with several studies, such as Ernberg et al., Oida et al., and Yıldırım et al. [11, 24, 26], all of which reported lower rates of DGE with SPGJ compared to CGJ. Moreover, Zhang et al. also reported a significant reduction in DGE, further supporting our findings [25]. The meta-analysis conducted by Kumagai et al. included seven studies and 207 patients evaluating the effectiveness of SPGJ compared to CGJ in patients with GOO. Their findings demonstrated that SPGJ significantly reduced the risk of DGE (RR = 0.32, 95% CI: 0.17–0.60) [27]. The meta-analysis by Lorusso et al. [9] further supported the benefits of SPGJ over CGJ, demonstrating significantly lower rates of DGE (OR = 4.9, 95% CI: 2.3–10.8). The reduction in DGE with SPGJ is likely due to its partitioning mechanism, which isolates the bypassed stomach from bile and pancreatic secretions, thus enhancing gastric emptying. The reduction in DGE with SPGJ is likely due to its partitioning mechanism, which isolates the bypassed stomach from bile and pancreatic secretions, thus enhancing gastric emptying. This mechanistic advantage may facilitate early postoperative recovery; however, in our pooled analysis it did not translate into clear reductions in length of stay or improved adherence to postoperative chemotherapy, indicating that the clinical impact of improved gastric emptying is primarily confined to the immediate postoperative period. Although SPGJ demonstrated consistently lower rates of DGE across studies, it is important to acknowledge that technical factors may also contribute to this difference. Variations in the extent of gastric partitioning, anastomotic configuration, surgeon experience, and perioperative management could influence gastric motility independently of the physiological effect of partitioning. Nonetheless, the effect was robust across heterogeneous settings and persisted in low-bias studies with minimal heterogeneity, suggesting that the reduced DGE is likely driven by both technical and physiological advantages inherent to the SPGJ procedure.

We did not find a significant difference in overall survival between SPGJ and CGJ (*p* = 0.82). Our findings are consistent with those of Ramos et al. (*p* = 0.15) and Hai et al. 2024 (*p* = 0.76) [7, 8]. Our results are consistent with the previous meta-analyses by Kumagai et al. and Lorusso et al. [9, 27] as all studies, including ours, did not find a statistically significant difference in overall survival between SPGJ and CGJ. We attempted to conduct a subgroup analysis focusing on different types of SPGJ to determine which surgical technique may be superior. However, none of the included studies reported outcomes stratified by specific surgical subtype. Instead, all studies presented only aggregated results across all techniques. Therefore, due to insufficient granular data, a meaningful subgroup analysis could not be performed. Our meta-analysis, which utilized IPD reconstruction from Kaplan-Meier curves, similarly failed to demonstrate a significant survival advantage. The lack of statistical significance may be partly explained by the limited sample sizes in the included studies, as most were not designed to detect differences in long-term survival but rather focused on primary outcomes such as GOOS. Additionally, important factors influencing OS include patients’ ability to maintain adequate oral intake and adherence to postoperative chemotherapy. In our study, there were no differences between the groups in these critical outcomes, which may explain the lack of a significant difference in OS. As reported by Ramos et al., improved survival was observed in patients achieving a GOOS score of 3 (*p* < 0.001) and those who received postoperative chemotherapy compared to those who did not (*p* < 0.001) [7]. Our IPD meta-analysis similarly failed to demonstrate a significant survival advantage for SPGJ over CGJ, which is consistent with prior meta-analyses by Kumagai et al. and Lorusso et al. that also reported no difference in overall survival. This absence of effect likely reflects the overriding influence of advanced tumor biology and systemic disease course in unresectable GOO, as well as the absence of differences in key determinants of oncologic outcome such as GOOS and chemotherapy adherence in our data. Taken together, the available evidence suggests that while SPGJ improves early gastric emptying and reduces complications, these benefits do not currently translate into proven survival gains.

SPGJ was associated with a lower risk of postoperative major complications compared to CGJ, which is consistent with the findings of Ramos et al. [7], Zhang et al. [25], Huang et al. [22], and Ernberg et al. [11]. These results also align with the previous meta-analysis by Kumagai [27], which reported reduced complication rates associated with SPGJ. The reduced incidence of complications may be attributed to enhanced gastric emptying achieved through the partitioned stomach structure, which effectively lowers intragastric pressure, minimizes anastomotic tension, and reduces the risk of leakage. From a technical perspective, the primary difference between SPGJ and CGJ is the partial division of the stomach using a linear stapler device. This straightforward modification is unlikely to increase surgical complication rates and may even contribute to a safer procedure. Furthermore, the improved gastric emptying associated with SPGJ may decrease the likelihood of postoperative complications by reducing the need for reinterventions over time, thereby enhancing overall patient outcomes.

Our meta-analysis demonstrated no significant differences between SPGJ and CGJ concerning several important safety and procedural outcomes, including the need for reintervention, short-term mortality, operation time, and length of hospital stay, which suggests that SPGJ is not inferior to CGJ in safety outcomes. Specifically, the need for reintervention was comparable between the two groups, indicating that the structural modification introduced by SPGJ does not compromise the long-term durability of the procedure. This finding aligns with studies by Abdel-lah-Fernández et al., Ernberg et al., Huang et al., and Yildirim [11, 20, 22, 26] which also reported similar reintervention rates between the two techniques.

Additionally, the absence of significant differences in short-term mortality suggests that the partial stomach partitioning involved in SPGJ does not increase the risk of perioperative death. This observation is further supported by the findings of Abdel-lah-Fernández et al., Ramos et al., Usuba et al., Yildirim, and Zhang et al. [7, 10, 20, 25, 26], which reported comparable survival rates during the early postoperative period for both techniques. The consistency of these findings across multiple studies enhances the credibility of SPGJ as a safe surgical option.

Operation time and length of hospital stay were also similar between SPGJ and CGJ, indicating that the additional step of partial stomach partitioning does not substantially prolong the surgical procedure or delay postoperative recovery. This observation is further supported by the findings of Usuba et al., Ramos et al., Huang et al., Oida et al., and Zhang et al. [7, 10, 22, 24, 25]. While SPGJ might be expected to increase operative time slightly due to the stapling process, the difference was not significant, suggesting that this modification does not impose a substantial burden on surgical efficiency.

Postoperative adherence to chemotherapy is a crucial outcome when evaluating the effectiveness of surgical interventions for GOO. Bypass surgery aims to improve patients’ clinical conditions, making them more suitable for receiving palliative chemotherapy, which is a critical factor associated with better overall survival. Our meta-analysis demonstrated that there was no significant difference in chemotherapy adherence between SPGJ and CGJ groups, aligning with previous findings by Ramos et al., Usuba et al., Abdel-lah-Fernández et al., and Kaminishi [7, 10, 20, 23].

### Strengths

Our meta-analysis addressed several limitations of the previous studies. Our meta-analysis included a total sample size of 456 patients, which is notably larger than the sample sizes of previous meta-analyses. Kumagai et al. 2016 included 207 patients from seven studies, while Lorusso et al. 2019 [9] included 226 patients from eight studies. The added number of patients in our meta-analysis is approximately 120.3% of the sample size used by Kumagai et al. 2016 and 101.8% of the sample size used by Lorusso et al. 2019 [9]. The larger sample size in our study provides greater statistical power, enhancing the reliability of our findings and allowing for a more accurate comparison of clinical outcomes between SPGJ and CGJ. Moreover, our study incorporates six recent studies not included in the previous meta-analyses [7, 8, 20, 22, 25, 26], thereby offering a more comprehensive assessment of the comparative efficacy and safety of SPGJ versus CGJ. Second, we utilized an IPD meta-analysis approach to reconstruct survival data from Kaplan-Meier curves, which provided a more precise estimation of overall survival compared to aggregate data methods used in previous meta-analyses. Additionally, we assessed important clinical outcomes that have not been reported previously. including GOOS score and adherence to postoperative chemotherapy, which has been identified as a critical factor influencing long-term survival. The previous studies included only retrospective studies, while our analysis pooled the most recent RCT published by Ramos et al. 2025 [7].

### Limitations

Despite the strengths of our meta-analysis, several limitations should be acknowledged. Because only one eligible RCT exists in the literature, this review necessarily relied on retrospective comparative studies, deviating from the initial PROSPERO plan. This limitation was addressed by applying the GRADE approach appropriate for observational evidence, including sensitivity analyses restricted to studies at low risk of bias. The heterogeneity observed across several outcomes may be partly explained by differences in study design. Among the included studies, only one was RCT, while the remaining ten were retrospective comparative studies. RCTs generally provide more rigorous control of confounding factors through randomization, whereas retrospective studies are more susceptible to selection bias, variations in baseline characteristics, and differences in perioperative management. These methodological discrepancies likely contributed to the variability in effect estimates, particularly for outcomes such as GOOS, operation time, and length of hospital stay, where substantial heterogeneity was detected. Although sensitivity analyses, including leave-one-out tests, reduced heterogeneity for some endpoints (e.g., delayed gastric emptying and operation time), the persistence of moderate to high heterogeneity in other outcomes indicates that study design differences and unmeasured confounders remain important sources of variability. An additional limitation is the inability to account for surgeon- and center-level learning-curve effects. Outcomes of gastrojejunostomy—particularly when performed laparoscopically—are strongly operator-dependent, yet none of the included studies reported surgeon experience, annual case volume, or institutional proficiency levels. As a result, unmeasured variation in technical expertise may have influenced the observed short-term outcomes. This limitation should be considered when interpreting the pooled results. Although the inclusion of the most recent RCT by Ramos et al. [7] adds robustness to our findings, but the overall quality of evidence remains limited. Additionally, the relatively small sample sizes in individual studies may have contributed to the lack of statistical power needed to detect significant differences in certain outcomes. While our total sample size is larger than that of previous meta-analyses, it may still be inadequate to detect subtle differences in some outcomes. A small proportion of patients with benign gastric outlet obstruction were included in some studies; however, as malignant cases constituted the overwhelming majority. Because no study provided outcome stratification by etiology and malignant cases represented the substantial majority, we retained these studies in accordance with Cochrane recommendations on mixed populations when separation is not feasible. Given the small proportion of benign cases and the malignant context of all included cohorts, this is unlikely to have materially influenced our findings. Key patient-centered outcomes such as quality of life and time to oral intake were seldom reported and were not available in a consistent format for synthesis. As a result, our analysis relied on DGE, GOOS, and major complications as functional surrogates, which remain highly relevant but do not fully capture the patient experience in unresectable malignant GOO.

Some of the included studies also demonstrated a risk of bias, which could affect the reliability of the pooled estimates. Moreover, publication bias could not be thoroughly assessed due to the limited number of studies in each analysis. Despite employing rigorous methodological approaches, our findings remain constrained by the inherent biases and weaknesses of the included studies. Future research should prioritize well-designed RCTs with larger sample sizes, standardized outcome definitions, and longer follow-up periods to provide more robust and definitive conclusions.

## Conclusion

SPGJ appears to reduce delayed gastric emptying and major postoperative complications compared with CGJ. However, these early advantages did not translate into clear improvements in length of stay, adherence to postoperative chemotherapy, or overall survival. High-quality randomized controlled trials with standardized outcome definitions and adequate statistical power are needed to confirm these findings and better inform clinical practice.

## Supplementary Information

Supplementary Material 1.

Supplementary Material 2.

Supplementary Material 3.

Supplementary Material 4.

Supplementary Material 5.

Supplementary Material 6.

Supplementary Material 7.

Supplementary Material 8.

## Data Availability

The reconstructed individual patient data (IPD) datasets generated from published Kaplan–Meier curves are available upon reasonable request.
