# Supplementary material for: Gastric partitioning compared to conventional gastrojejunostomy as palliative surgeries in patients with gastric outlet obstruction: a pairwise and individual patient data meta-analysis
Source: World J Surg Oncol. 2026 Jan 13;24:56. doi: 10.1186/s12957-025-04166-6 (PMC12838411; doi:10.1186/s12957-025-04166-6)
Supplement: Supplementary file 1 — Supplementary Material 1. [file 12957_2025_4166_MOESM1_ESM.docx]

| Supplementary Table 1. Search strategy for each database | | |
| --- | --- | --- |
| Database | **Search strategy** | **Number of results** |
| PubMed | (("Gastric Outlet Obstruction"[Mesh] OR "duodenal obstruction" OR  "pyloric obstruction" OR "gastric obstruction" OR "GOO") AND  ("Gastrojejunostomy"[Mesh] OR gastrojejunostomy OR gastroenterostomy OR  gastrointestinal bypass OR gastric bypass) AND  ("stomach partitioning" OR "stomach-partitioning" OR "Devine procedure" OR  "modified Devine" OR partitioning)) | 42 |
| Scopus | (("Gastric Outlet Obstruction" OR "duodenal obstruction" OR  "pyloric obstruction" OR "gastric obstruction" OR "GOO") AND  ("Gastrojejunostomy" OR gastrojejunostomy OR gastroenterostomy OR  gastrointestinal bypass OR gastric bypass) AND  ("stomach partitioning" OR "stomach-partitioning" OR "Devine procedure" OR  "modified Devine" OR partitioning)) | 38 |
| Web of Science | (("Gastric Outlet Obstruction" OR "duodenal obstruction" OR  "pyloric obstruction" OR "gastric obstruction" OR "GOO") AND  ("Gastrojejunostomy" OR gastrojejunostomy OR gastroenterostomy OR  gastrointestinal bypass OR gastric bypass) AND  ("stomach partitioning" OR "stomach-partitioning" OR "Devine procedure" OR  "modified Devine" OR partitioning)) | 52 |
| Cochrane CENTRAL | (("Gastric Outlet Obstruction" OR "duodenal obstruction" OR  "pyloric obstruction" OR "gastric obstruction" OR "GOO") AND  ("Gastrojejunostomy" OR gastrojejunostomy OR gastroenterostomy OR  gastrointestinal bypass OR gastric bypass) AND  ("stomach partitioning" OR "stomach-partitioning" OR "Devine procedure" OR  "modified Devine" OR partitioning)) | 6 |
| ClinicalTrials.gov | (("Gastric Outlet Obstruction" OR "duodenal obstruction" OR  "pyloric obstruction" OR "gastric obstruction" OR "GOO") AND  ("Gastrojejunostomy" OR gastrojejunostomy OR gastroenterostomy OR  gastrointestinal bypass OR gastric bypass) AND  ("stomach partitioning" OR "stomach-partitioning" OR "Devine procedure" OR  "modified Devine" OR partitioning)) | 0 |
