# Supplementary figures and images for: Gastric partitioning compared to conventional gastrojejunostomy as palliative surgeries in patients with gastric outlet obstruction: a pairwise and individual patient data meta-analysis
Source: World J Surg Oncol. 2026 Jan 13;24:56. doi: 10.1186/s12957-025-04166-6 (PMC12838411; doi:10.1186/s12957-025-04166-6)

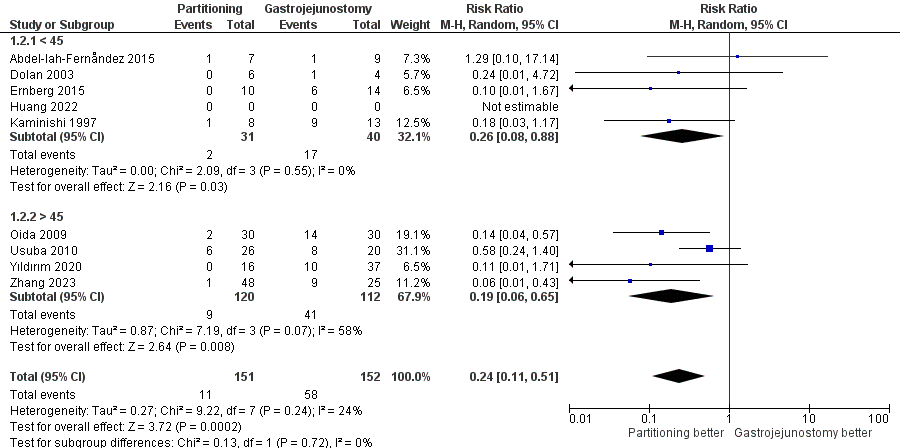

Supplement: Supplementary file 3 — Supplementary Material 3. [file 12957_2025_4166_MOESM3_ESM.png]

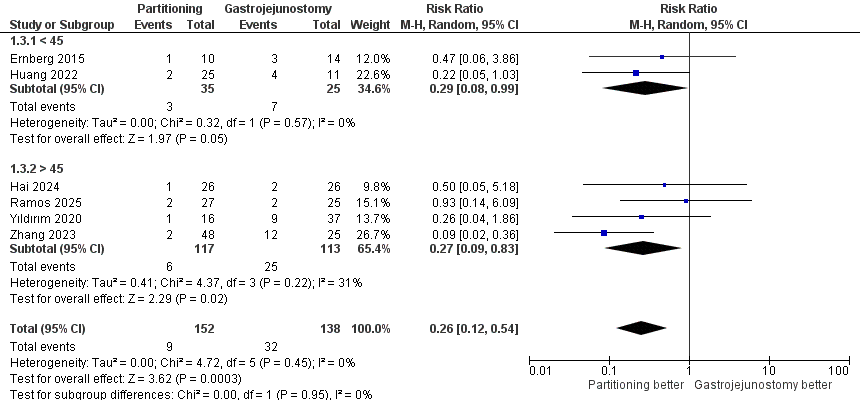

Supplement: Supplementary file 4 — Supplementary Material 4. [file 12957_2025_4166_MOESM4_ESM.png]

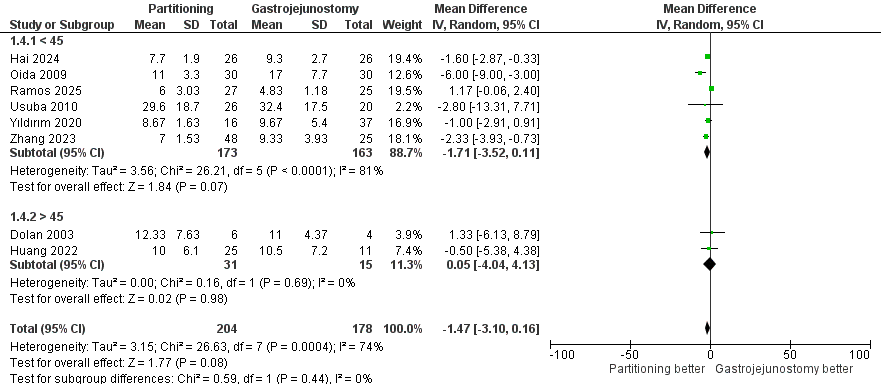

Supplement: Supplementary file 5 — Supplementary Material 5. [file 12957_2025_4166_MOESM5_ESM.png]

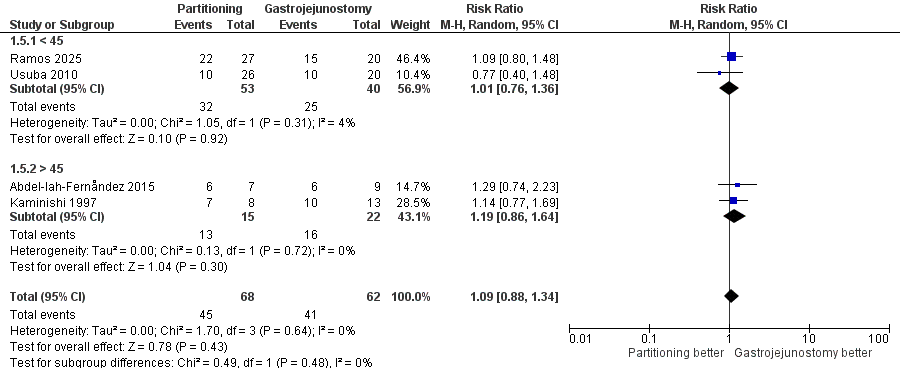

Supplement: Supplementary file 6 — Supplementary Material 6. [file 12957_2025_4166_MOESM6_ESM.png]

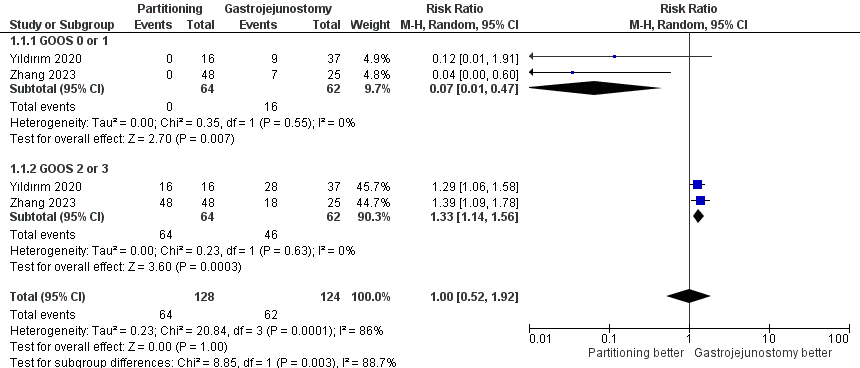

Supplement: Supplementary file 7 — Supplementary Material 7. [file 12957_2025_4166_MOESM7_ESM.png]

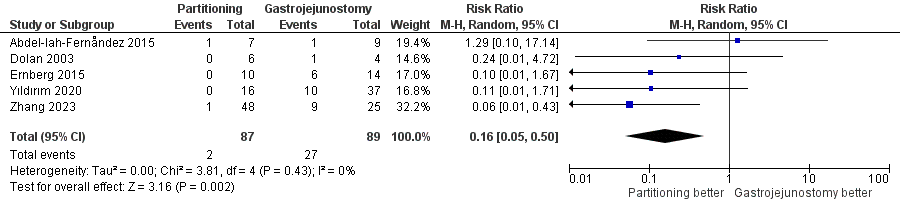

Supplement: Supplementary file 8 — Supplementary Material 8. [file 12957_2025_4166_MOESM8_ESM.png]
